# The Involvement of GAS6 Signaling in the Development of Obesity and Associated Inflammation

**DOI:** 10.1155/2015/202513

**Published:** 2015-04-14

**Authors:** Kuo-Sheng Wu, Yi-Jen Hung, Chien-Hsing Lee, Fone-Ching Hsiao, Po-Shiuan Hsieh

**Affiliations:** ^1^Department of Physiology and Biophysics, National Defense Medical Center, Taipei 114, Taiwan; ^2^Division of Endocrinology and Metabolism, Department of Internal Medicine, Tri-Service General Hospital, National Defense Medical Center, Taipei 114, Taiwan; ^3^Institute of Preventive Medicine, National Defense Medical Center, 114 Taipei, Taiwan

## Abstract

Growth arrest-specific 6 (GAS6), a vitamin K-dependent protein, plays a role in the survival, proliferation, migration, differentiation, adhesion, and apoptosis of cells. GAS6 is highly expressed during growth arrest, followed by a sharp decrease during differentiation in adipocytes. The functions of GAS6 signaling are limited to TAM (Tyro3, Axl, and Mer) receptors and are dependent on the cell type. While many studies have focused on the role of GAS6 in inflammation and cancer, only few studies focused on its roles of GAS6 in obesity. Accordingly, the participation of GAS6 in the progression of obesity remains controversial. In this review, we summarize the results of current studies from clinical and basic research to elucidate the possible role of GAS6 signaling in obesity and associated disorders. In addition, this summary may offer a direction to develop clinical therapeutic strategies for the prevention and treatment of obesity and related complications.

## 1. Introduction

Growth arrest-specific 6 (GAS6) was identified in 1988 and was further characterized in mouse embryonic NIH 3T3 fibroblasts in 1993 [1, 2]. It is considered a growth arrest protein involved in regulation of the cell cycle. To date, GAS6 has been reported to be involved in the processes of proliferation, differentiation, and inflammation in various cell types (adipocytes, endothelial cells, vascular smooth muscle cells, bone marrow cells, etc.) and tissues (ovary, heart, kidney, etc.) [2–4]. It is worth mentioning that the role of GAS6 signaling in inflammation remains controversial nowadays. In dendritic cells and macrophages, it negatively regulates inflammation, but it promotes phagocytosis of apoptotic cells and also positively participates in the maturation of natural killer cells [5].

GAS6 is a member of the vitamin K-dependent protein family, and it contains 678-amino acids. GAS6 shares approximately 42% similarity with protein S, a nonenzymatic cofactor for activated protein C and its downstream cascades leading to the inhibition of coagulation [6]. Although the GAS6 protein shares structural homology with protein S, their functions are dissimilar. The functions of GAS6 are limited to binding with TAM (Tyro3, Axl, and Mer) receptors, particularly with Axl. GAS6 shows the highest affinity to bind to Axl, followed by Tyro3 and lastly to Mer [7]. GAS6 interacts with TAM receptors through its SHBG-domain to activate downstream signaling pathways, such as PLC*γ*, PI3K, ERK, and NF-*κ*B to regulate cell survival, proliferation, migration, differentiation, adhesion, and apoptosis [8, 9]. Analysis of GAS6 and TAM receptors signaling is likely to be fundamental to improve the understanding of disease progression and development of therapeutics.

Obesity has been shown to be a predominant risk factor in the progression of chronic cardiometabolic disorders, such as diabetes, chronic renal failure, cardiovascular disease, and hypertension [10]. However, only a few reports have been published regarding the link between GAS6 signaling and the pathogenesis of obesity. This paper summarizes the relevant clinical and basic researches published to date and attempts to pinpoint the potential role of GAS6 signaling molecules in the development of obesity and associated inflammation.

## 2. GAS6 Signaling in the Development of Obesity

The recent studies focusing on GAS6 signaling and the development of obesity in various studies from clinical and basic researches are summarized in Tables 1 and 2. For instance, a recent clinical study conducted in Taiwan teenagers from our research team showed that levels of circulating GAS6 (12.3 versus 13.9 ng/mL) and soluble Axl (3.8 versus 4.7 ng/mL) were significantly increased in overweight and obese adolescents and were positively correlated with BMI (19.5 versus 27.8 kg/m^2^) and body fat mass (Table 2) [11]. Furthermore, we also analyzed genetic polymorphisms among Taiwan adolescents and the result further showed an increased BMI and waist circumference significantly correlated with the GG genotype of GAS6 (rs8191974) [12]. This genotyping of GAS6 might be suitable for predicting the future progress of obesity and its related complications.

Consistently, GAS6 expression was increased in subcutaneous fat of high-fat fed mice, compared to those fed a standard fat diet (363 versus 976 copy number) (Table 1) [13]. In addition, GAS6-deficient mice exhibited significantly less fat accumulation in the subcutaneous and gonadal fat pads than wild-type mice when fed a high-fat diet (Table 1) [3]. From mechanistic aspect, the* in vitro* study showed that GAS6 was highly expressed at growth arrest and participated in cell proliferation and differentiation [2, 14].

In addition, studies showed that GAS6 receptor-Axl might have a role in the development of obesity. A report demonstrated that mice overexpressing Axl showed an increase in body weight gain and may subsequently cause the development of obesity (Table 1) [15]. In addition, antagonizing the Axl receptor with the administration of R428 by oral gavage impaired adipose tissue development through inhibition of preadipocyte differentiation into mature adipocytes that resulted from reduced lipid uptake in mice [16]. The R428 treatment significantly reduced body weight gain and also subcutaneous (831 versus 421 mg) and gonadal (123 versus 685 mg) fat mass after high-fat diet feeding compared to controls (Table 1). The expression levels of GAS6 (3.11 versus 2.58 (ΔCT)) and Mer (6.74 versus 6.17 (ΔCT)) showed a significant decrease in gonadal fat but not in subcutaneous fat in R428-treated mice (Table 1). Collectively, these observations implicate that GAS6 may participate in adipogenesis by regulating cell proliferation and differentiation through TAM receptors and may subsequently affect the development of obesity.

Nevertheless, the clinical study conducted with elderly patients with type 2 diabetes showed that plasma GAS6 level (14.3 versus 11.5 ng/mL) was negatively correlated with BMI (23.9 versus 26 kg/m^2^) and blood glucose level, compared to healthy individuals (Table 2) [17, 18], implying that aging and hyperglycemia might be the significant cofounding factors that affect circulating GAS6 levels. Moreover, as shown in Table 2, the plasma GAS6 level did not differ between overweight males and females [19]. However, plasma GAS6 levels were significantly lowered in postmenopausal than in premenopausal overweight females, implying a potential effect of estrogen on GAS6 expression [20]. These observations implicate that the GAS6 expression level is accounted not only for obesity but also for other factors such as ages, sex hormones, and hyperglycemia.

On the other hand, there was a contradictory result from the animal study suggesting that Axl deficiency had no significant effect on adipogenesis [13]. They found that deficiency of Axl in mouse embryonic fibroblasts did not affect their differentiation and lipid uptake. Furthermore, the body weight gain, subcutaneous and gonadal fat mass, and GAS6 signaling molecules showed no significant differences between Axl-deficient or wild-type mice fed on either a standard diet or a high-fat diet (Table 1) [13]. However, compared to standard fat diet feeding, Axl-deficient mice fed a high-fat diet showed a dramatic increase in the mRNA expression levels of Mer (74 versus 147 copy number) and Tyro3 (5.1 versus 13 copy number) in subcutaneous adipose tissues (Table 1) [13]. This compensatory enhancement of genes expression caused by high-fat diet feeding was not observed in wild-type mice. Based on this finding, the authors speculated that the other two TAM receptors (Mer and Tyro3) compensate for the effect of Axl deficiency in GAS6 signaling [13]. Since Axl-deficient mice show that their Axl mRNA levels were increased in mice fed a high-fat diet compared to a standard fat diet (102 versus 371 copy number) and a similar trend was observed in the wild-type group in subcutaneous adipose tissues (420 versus 1127 copy number) (Table 1), the authors speculated that the Axl mRNA levels detected in Axl-deficient mice might be the incomplete fragments with no function to transduce signaling. However, it still has another possibility that Axl did not knockout completely in their mouse model, and its low expression levels still can transduce GAS6 signaling. Taken together, GAS6 signaling is important for the development of obesity, but the underlying mechanism remains to be elucidated.

## 3. GAS6 Signaling in Obesity-Associated Inflammation

Obesity is considered a chronic inflammatory disease. Plasma and adipose tissue levels of inflammatory cytokines such as IL-6 and TNF-*α* have been reported to increase along with the progression of obesity [21, 22]. In a clinical study, circulating GAS6 and soluble Axl levels were found to be positively correlated with plasma TNF-*α*, IL-6, and C reactive protein (CRP) levels in overweight and obese adolescents (Table 4) [11]. Furthermore, the CRP level was significantly elevated in boys carrying the GG polymorphism of the GAS6 rs8191974 genotype [12]. These observations imply that circulating GAS6 and soluble Axl levels are positively correlated with obesity-induced inflammation in adolescents, especially in boys. In addition, females show a lower correlation between GAS6 expression and systemic inflammation indicated by the increase in plasma CRP levels [19], which might be attributed to the effect of estrogen in the link between GAS6 expression and inflammation and it requires further investigation.

On the other hand, a previous study showed that elderly patients with type 2 diabetes exhibited a significantly decreased GAS6 level but a significantly increased inflammatory marker-plasma CRP level compared to healthy individuals [17, 18], suggesting that the relationship between circulating GAS6 level and obesity-induced inflammation could be significantly affected by ageing and hyperglycemia.

Furthermore, Axl, an important receptor of GAS6, originally characterized as a transforming gene in human myeloid leukemia cells, has been shown to play a role in regulating inflammation [23]. The Axl transgenic mice exhibited phenotypes of obesity and type 2 diabetes, such as hyperglycemia, hyperinsulinemia, and insulin resistance, and showed elevated plasma TNF-*α* level (Table 3) [15].

Collectively, these findings indicate that the correlation between GAS6 and systemic inflammation could be significantly affected by ageing, gender, and hyperglycemia. The Axl receptor-mediated pathway may play a substantial role in GAS6-mediated inflammatory signaling in states of obesity and associated type 2 diabetes.

## 4. GAS6 Signaling in the Clinical Implications for Obesity Treatment

The normal plasma concentration of GAS6 is in the range of 20–50 ng/mL, and patients with cancer or inflammation showed higher concentrations. In addition, GAS6 signaling has been demonstrated to be associated with various chronic cardiometabolic disorders, including thrombosis, diabetes, chronic renal failure, cardiovascular disease, and cancer [6, 24–27]. GAS6 expression was increased in subcutaneous fat in high-fat feeding mice [13]. In addition, GAS6-deficient mice exhibited significantly less fat accumulation in the subcutaneous and gonadal fat [3]. The level of circulating GAS6 was positively correlated with BMI and inflammatory cytokines in adolescents [11]. These observations suggest that GAS6 might be casually correlated with the obesity and associated inflammation. However, this correlation could be overridden under conditions of hyperglycemia and ageing.

Furthermore, GAS6 is the only ligand known to induce Axl downstream cascades among TAM receptors. Like GAS6, the level of plasma Axl was positively correlated with BMI and inflammatory cytokines in adolescents [11]. Administration of R428 reduced lipid uptake resulting in decreasing subcutaneous and gonadal fat mass and also body weight gain in mice [16]. It is implicated that GAS6 receptor Axl might be a top priority for the development of therapeutic strategies in obesity and related complications.

## 5. Conclusion

Of about 650 papers on growth arrest-specific genes were published since GAS6 was first cloned in 1988, more than 400 have focused on GAS6 signaling. Several recent clinical studies have shown that GAS6 signaling significantly contributes to the development of obesity and associated inflammation. In particular, GAS6/Axl signaling is important in the mechanism underlying inflammation that resulted from obesity and associated complications. However, the causal relationship between GAS6 and obesity-related cardiometabolic abnormalities remains controversial. From a clinical perspective, clarifying the role of GAS6 signaling from bench to clinic will provide great benefit in development of strategies for the prevention and treatment of obesity and its related complications.

## Figures and Tables

**Table 1:** GAS6 signaling molecules and the development of obesity in mouse model.

Model	Comparison	Treatment	GAS6 (tissue)	Axl (tissue)	Mer (tissue)	Tyro3 (tissue)	Body weight (g)	SC fat weight (mg)	GON fat weight (mg)	References
Axl+/+ mice	Axl+/+ versus WT						↑ (Axl+/+)			Augustine et al., 1999 [15]

GAS6−/− mice	GAS6−/− versus WT		— (GAS6−/−) (SC and GON)				NS	↓ (GAS6−/−)	↓ (GAS6−/−)	Maquoi et al., 2005 [3]

GAS6−/− mice	GAS6−/− versus WT		— (GAS6−/−) (vaginas)	↑ (GAS6−/−) (vaginas)	↑ (GAS6−/−) (vaginas)	↑ (GAS6−/−) (vaginas)				Salian-Mehta et al., 2014 [28]

C57BL/6 mice	Vehicle versus R428	R428 oral	4.3 ± 0.2 versus 4.1 ± 0.3 (ΔCT) (SC)	4.5 ± 0.2 versus 4.5 ± 0.2 (ΔCT) (SC)	7.5 ± 0.3 versus 7.2 ± 0.2 (ΔCT) (SC)	10.7 ± 0.3 versus 10.4 ± 0.3 (ΔCT) (SC)	30.3 ± 0.7 versus 25.3 ± 0.7^∗^	831.0 ± 58.0 versus 421.0 ± 69.0^∗^	123.0 ± 263.0 versus 685.0 ± 92.0^∗^	Lijnen et al., 2011 [16]

C57BL/6 mice	Vehicle versus R428	R428 oral	3.1 ± 0.1 versus 2.6 ± 0.1^∗^ (ΔCT) (GON)	3.9 ± 0.1 versus 3.6 ± 0.1 (ΔCT) (GON)	6.7 ± 0.2 versus 6.2 ± 0.2^∗^ (ΔCT) (GON)	10.0 ± 0.2 versus 10.1 ± 0.1 (ΔCT) (GON)	30.3 ± 0.7 versus 25.3 ± 0.7^∗^	831.0 ± 58.0 versus 421.0 ± 69.0^∗^	123.0 ± 263.0 versus 685.0 ± 92.0^∗^	Lijnen et al., 2011 [16]

Axl−/− mice	Axl−/− versus WT	SFD	321.0 ± 59.0 versus 363.0 ± 77.0 (CN) (SC)	102.0 ± 20.0 versus 420.0 ± 109.0 (CN) (SC)	74.0 ± 24.0 versus 83.0 ± 16.0 (CN) (SC)	5.1 ± 1.0 versus 7.6 ± 1.5 (CN) (SC)	22.0 ± 0.5 versus 22.0 ± 0.5	183.0 ± 23.0 versus 182.0 ± 9.6		Scroyen et al., 2012 [13]

Axl−/− mice	Axl−/− versus WT	SFD	1776.0 ± 85.0 versus 1777.0 ± 105.0 (CN) (GON)	362.0 ± 80.0 versus 1278.0 ± 102.0 (CN) (GON)	227.0 ± 24.0 versus 225.0 ± 24.0 (CN) (GON)	13.0 ± 0.9 versus 15.0 ± 1.1 (CN) (GON)	22.0 ± 0.5 versus 22.0 ± 0.5		249.0 ± 44.0 versus 253.0 ± 19.0	Scroyen et al., 2012 [13]

Axl−/− mice	Axl−/− versus WT	HFD	1135.0 ± 57.0 versus 976.0 ± 106.0 (CN) (SC)	371.0 ± 97.0 versus 1127.0 ± 169.0 (CN) (SC)	147.0 ± 14.0 versus 126.0 ± 18.0 (CN) (SC)	13.0 ± 1.2 versus 12.0 ± 1.1 (CN) (SC)	30.0 ± 1.2 versus 30.0 ± 1.3	888.0 ± 92.0 versus 781.0 ± 96.0		Scroyen et al., 2012 [13]

Axl−/− mice	Axl−/− versus WT	HFD	1193.0 ± 124.0 versus 1181.0 ± 111.0 (CN) (GON)	299.0 ± 79.0 versus 1168.0 ± 128.0 (CN) (GON)	187.0 ± 30.0 versus 166.0 ± 19.0 (CN) (GON)	12 ± 0.9 versus 14 ± 1.1 (CN) (GON)	30.0 ± 1.2 versus 30.0 ± 1.3		1476.0 ± 165.0 versus 1160.0 ± 120.0	Scroyen et al., 2012 [13]

^∗^ *P* < 0.05; R428, Axl receptor antagonist; SC, subcutaneous fat; GON, gonadal fat; HFD, high-fat diet; SFD, standard fat diet; NS, No significant difference; and CN, copy number.

**Table 2:** GAS6 signaling molecules and the development of obesity in clinical studies.

Model	Comparison	Age	GAS6 (plasma, ng/mL)	Axl (plasma, ng/mL)	BMI (kg/m^2^)	References
T2DM	Normal versus T2DM	50.2 ± 1.5 versus 52.4 ± 1.5	14.3 ± 0.7 versus 11.5 ± 0.4^∗^		23.9 ± 0.4 versus 26.0 ± 0.4^∗^	Lee et al., 2012 [26], Hung et al., 2010 [17]

T2DM	Normal versus T2DM	51.3 ± 1.5 versus 53.8 ± 1.3^∗^	15.2 ± 0.4 versus 11.2 ± 0.3^∗^			Lee et al., 2014 [29]

Human	Male versus female	49.1 ± 1.5 versus 55.8 ± 0.1^∗^	12.6 ± 0.5 versus 13.4 ± 0.5		24.8 ± 0.3 versus 25.4 ± 0.4	Kuo et al., 2014 [19]

Female	Pre- versus postmenopausal	34.1 ± 10.7 versus 58.9 ± 7.1^∗^	21.8 ± 8.9 versus 18.6 ± 9.4^∗^		25.3 ± 3.7 versus 25.2 ± 3.9	Hung et al., 2014 [20]

Adolescents	Lean versus obesity	13.3 ± 0.9 versus 13.2 ± 1.0	12.3 ± 4.4 versus 13.9 ± 3.9^∗^	3.8 ± 1.0 versus 4.7 ± 1.3^∗^	19.5 ± 1.5 versus 27.8 ± 2.7^∗^	Hsiao et al., 2013 [11]

Boys with GAS6 rs8191974 genotype	GG versus AA		13.1 ± 3.7 versus 13.0 ± 3.1		22.5 ± 4.2 versus 20.7 ± 3.3^∗^	Hsiao et al., 2014 [12]

^∗^ *P* < 0.05. T2DM: type 2 diabetes mellitus patients.

**Table 3:** GAS6 signaling molecules and the development of obesity-associated inflammation in mouse model.

Model	Comparison	Treatment	GAS6 (tissue)	Axl (tissue)	Mer (tissue)	Tyro3 (tissue)	TNF-*α* (plasma)	References
Axl+/+ mice	Axl+/+ versus WT						↑ (Axl+/+)	Augustine et al., 1999 [15]

GAS6−/− mice	GAS6−/− versus WT		— (GAS6−/−) (SC and GON)					Maquoi et al., 2005 [3]

GAS6−/− mice	GAS6−/− versus WT		— (GAS6−/−) (vaginas)	↑ (GAS6−/−) (vaginas)	↑ (GAS6−/−) (vaginas)	↑ (GAS6−/−) (vaginas)		Salian-Mehta et al., 2014 [28]

C57BL/6 mice	Vehicle versus R428	R428 oral	4.3 ± 0.2 versus 4.1 ± 0.3 (ΔCT) (SC)	4.5 ± 0.2 versus 4.5 ± 0.2 (ΔCT) (SC)	7.5 ± 0.3 versus 7.2 ± 0.2 (ΔCT) (SC)	10.7 ± 0.3 versus 10.4 ± 0.3 (ΔCT) (SC)		Lijnen et al., 2011 [16]

C57BL/6 mice	Vehicle versus R428	R428 oral	3.1 ± 0.1 versus 2.6 ± 0.1^∗^ (ΔCT) (GON)	3.9 ± 0.1 versus 3.6 ± 0.1 (ΔCT) (GON)	6.7 ± 0.2 versus 6.2 ± 0.2^∗^ (ΔCT) (GON)	10.0 ± 0.2 versus 10.1 ± 0.1 (ΔCT) (GON)		Lijnen et al., 2011 [16]

Axl−/− mice	Axl−/− versus WT	SFD	321.0 ± 59.0 versus 363.0 ± 77.0 (CN) (SC)	102.0 ± 20.0 versus 420.0 ± 109.0 (CN) (SC)	74.0 ± 24.0 versus 83.0 ± 16.0 (CN) (SC)	5.1 ± 1.0 versus 7.6 ± 1.5 (CN) (SC)		Scroyen et al., 2012 [13]

Axl−/− mice	Axl−/− versus WT	SFD	1776.0 ± 85.0 versus 1777.0 ± 105.0 (CN) (GON)	362.0.0 ± 80 versus 1278.0 ± 102.0 (CN) (GON)	227.0 ± 24.0 versus 225.0 ± 24.0 (CN) (GON)	13.0 ± 0.9 versus 15.0 ± 1.1 (CN) (GON)		Scroyen et al., 2012 [13]

Axl−/− mice	Axl−/− versus WT	HFD	1135.0 ± 57.0 versus 976.0 ± 106.0 (CN) (SC)	371.0 ± 97.0 versus 1127.0 ± 169.0 (CN) (SC)	147.0 ± 14.0 versus 126.0 ± 18.0 (CN) (SC)	13.0 ± 1.2 versus 12.0 ± 1.1 (CN) (SC)		Scroyen et al., 2012 [13]

Axl−/− mice	Axl−/− versus WT	HFD	1193.0 ± 124.0 versus 1181.0 ± 111.0 (CN) (GON)	299.0 ± 79.0 versus 1168.0 ± 128.0 (CN) (GON)	187.0 ± 30.0 versus 166.0 ± 19.0 (CN) (GON)	12 ± 0.9 versus 14 ± 1.1 (CN) (GON)		Scroyen et al., 2012 [13]

^∗^ *P* < 0.05; R428, Axl receptor antagonist; SC, subcutaneous fat; GON, gonadal fat; HFD, high-fat diet; SFD, standard fat diet; and CN, copy number.

**Table 4:** GAS6 signaling molecules and the development of obesity-associated inflammation in clinical studies.

Model	Comparison	Age	GAS6 (plasma, ng/mL)	Axl (plasma, ng/mL)	TNF-*α* (plasma, ng/mL)	IL-6 (plasma, pg/mL)	hsCRP (plasma, mg/L)	BMI (kg/m^2^)	References
T2DM	Normal versus T2DM	50.2 ± 1.5 versus 52.4 ± 1.5	14.3 ± 0.7 versus 11.5 ± 0.4^∗^		3.2 ± 0.2 versus 3.3 ± 0.2	2.6 ± 0.5 versus 5.9 ± 1.4	0.7 ± 0.1 versus 1.2 ± 0.1^∗^	23.9 ± 0.4 versus 26.0 ± 0.4^∗^	Lee et al., 2012 [26], Hung et al., 2010 [17]

T2DM	Normal versus T2DM	51.3 ± 1.5 versus 53.8 ± 1.3^∗^	15.2 ± 0.4 versus 11.2 ± 0.3^∗^						Lee et al., 2014 [29]

Human	Male versus female	49.1 ± 1.5 versus 55.8 ± 0.1^∗^	12.6 ± 0.5 versus 13.4 ± 0.5		3.2 ± 0.2 versus 3.3 ± 0.2	4.7 ± 1.0 versus 4.2 ± 1.0	0.8 ± 0.1 versus 1.1 ± 0.1^∗^	24.8 ± 0.3 versus 25.4 ± 0.4	Kuo et al., 2014 [19]

Female	Pre- versus postmenopausal	34.1 ± 10.7 versus 58.9 ± 7.1^∗^	21.8 ± 8.9 versus 18.6 ± 9.4^∗^					25.3 ± 3.7 versus 25.2 ± 3.9	Hung et al., 2014 [20]

Adolescents	13.3 ± 0.9 versus 13.2 ± 1.0	13.3 ± 0.9 versus 13.2 ± 1.0	12.3 ± 4.4 versus 13.9 ± 3.9^∗^	3.8 ± 1.0 versus 4.7 ± 1.3^∗^	14.0 ± 3.0 versus 62.9 ± 15.9^∗^	3.0 ± 0.1 versus 3.5 ± 5.8^∗^	0.6 ± 1.1 versus 1.0 ± 1.2^∗^	19.5 ± 1.5 versus 27.8 ± 2.7^∗^	Hsiao et al., 2013 [11]

Boys with gas6 rs8191974 genotype	GG versus AA		13.1 ± 3.7 versus 13.0 ± 3.1		26.7 ± 8.2 versus 23.8 ± 4.6	3.4 ± 2.8 versus 2.5 ± 1.3	0.8 ± 1.2 versus 0.4 ± 0.2^∗^	22.5 ± 4.2 versus 20.7 ± 3.3^∗^	Hsiao et al., 2014 [12]

^∗^ *P* < 0.05. T2DM: type 2 diabetes mellitus patients.
